# Editorial: Artificial Intelligence and Human Movement in Industries and Creation

**DOI:** 10.3389/frobt.2021.712521

**Published:** 2021-07-12

**Authors:** Kosmas Dimitropoulos, Petros Daras, Sotiris Manitsaris, Frederic Fol Leymarie, Sylvain Calinon

**Affiliations:** ^1^ Information Technologies Institute, Centre for Research and Technology Hellas, Thessaloniki, Greece; ^2^ Centre for Robotics, MINES ParisTech, PSL Université Paris, Paris, France; ^3^ Department of Computing, Goldsmiths University of London, London, United Kingdom; ^4^ Idiap Research Institute, Martigny, Switzerland

**Keywords:** Artificial intelligence, human motion analysis, human centred, machine learning, motion caption

Recent advances in human motion sensing technologies and machine learning have enhanced the potential of Artificial Intelligence to improve our quality of life, increase productivity and reshape multiple industries, including cultural and creative industries. In order to achieve this goal, humans must remain at the center of Artificial Intelligence and AI should learn from humans and collaborate effectively with them. Human-Centred Artificial Intelligence (HAI) is expected to create new opportunities and challenges in the future, which cannot yet be foreseen. Any type of programmable entity (e.g., robots, computers, autonomous vehicles, drones, Internet of Things, etc.) will have different layers of perception and sophisticated HAI algorithms that will detect human intentions and behaviors (Psaltis et al., 2017) and learn continuously from them. Thus, every single intelligent system will be able to capture human motions, analyze them (Zhang et al., 2019), detect poses and recognize gestures (Chatzis et al., 2020; Stergioulas et al., 2021) and activities (Papastratis et al., 2020; Papastratis et al., 2021; Konstantinidis et al., 2021), including facial expressions and gaze (Bek et al., 2020), enabling natural collaboration with humans.

Different sensing technologies, such as optical Mocap systems, wearable inertial sensors, RGB or depth cameras and other modality type sensors, are employed for capturing human movement in the scene and transforming this information into a digital representation. Most of the researchers usually focus on the use of a single modality sensor - due to the simplicity and low cost of the final system - and the design of either conventional machine learning algorithms or complex deep learning network architectures for analyzing human motion data (Konstantinidis et al., 2018; Konstantinidis et al., 2020). Such cost-effective approaches have been applied to a wide range of application domains, including entertainment (Kaza et al., 2016; Baker, 2020), health (Dias et al.; Konstantinidis et al., 2021), education (Psaltis et al., 2017; Stefanidis et al., 2019), sports (Tisserand et al., 2017), robotics (Jaquier et al., 2020; Gao et al., 2021), art and cultural heritage (Dimitropoulos et al., 2018), showing the great potential of AI technology.

Based on the aforementioned, it is evident that HAI is currently at the center of scientific debates and technological exhibitions. Developing and deploying intelligent machines is definitely both an economic challenge (e.g., flexibility, simplification, ergonomy) as well as a societal challenge (e.g., safety, transparency), not only from a factory perspective, but also for the real-world in general. The papers in this Research Topic adopt different sensing technologies, such as depth sensors, inertial suits, IMU sensors and force-sensing resistors (FSRs) to capture human movement, while they present diverse approaches for modeling the temporal data.

More specifically, Sakr et al. investigate the feasibility of employing FSRs worn on the arm to measure the Force Myography (FMG) signals for isometric force/torque estimation. A two-stage regression strategy is employed to enhance the performance of the FMG bands, where three regression algorithms including general regression neural network (GRNN), support vector regression (SVR), and random forest regression (RF) models are used, respectively, in the first stage, while GRNN is used in the second stage. Two cases are considered to explore the performance of the FMG bands in estimating: (a) 3-DoF force and 3-DoF torque at once and (b) 6-DoF force and torque. In addition, the impact of sensor placement and the spatial coverage of FMG measurements is studied.

Manitsaris et al. propose a multivariate time series approach for the recognition of professional gestures and for the forecasting of their trajectories. More specifically, the authors introduce a gesture operational model, which describes how gestures are performed based on assumptions that focus on the dynamic association of body entities, their synergies, and their serial and non-serial mediations, as well as their transitioning over time from one state to another. The assumptions of this model are then translated into an equation system for each body entity through State-Space modeling. The proposed method is evaluated on four industrial datasets that contain gestures, commands and actions.

A comprehensive review on machine learning approaches for motor learning is presented by Caramiaux et al. The review outlines existing machine learning models for motor learning and their adaptation capabilities and identifies three types of adaptation: Parameter adaptation in probabilistic models, transfer and meta-learning in deep neural networks, and planning adaptation by reinforcement learning.

Dias et al. present an innovative and personalized motor assessment tool capable of monitoring and tracking the behavioral change of Parkinson’s disease (PD) patients (mostly related to posture, walking/gait, agility, balance, and coordination impairments). The proposed assessment tool is part of the i-Prognosis Game Suit, which was developed within the framework of the i-Prognosis EU funded project (www.i-prognosis.eu). Six different motor assessments tests integrated in the iPrognosis Games have been designed and developed based on the UPDRS Part III examination. The efficiency of the proposed assessment tests to reflect the motor skills status, similarly to the UPDRS Part III items, is validated via 27 participants with early and moderate PD.

Bikias et al. explore the use of IMU sensors for the detection of Freezing-of-Gait (FoG) Episodes in Parkinson’s disease Patients and present a novel deep learning method. The study investigates the feasibility of a single wrist-based inertial measurement unit (IMU) for effectively predicting FoG events. The proposed method, namely, DeepFoG, aims at facilitating the real-time detection of FoG episodes. DeepFoG is based on the training of a deep learning model that automatically detects FoG events and differentiates them from stops and walking with turns. DeepFoG, utilizing a single-arm sensor has the potential to achieve similar accuracy as previously published methods, but with fewer sensors. The main advantage offered by the proposed methodology is its simplification and convenience attributed to the use of a single smartwatch rather than its improved accuracy.

The approaches discussed in this Research Topic offer readers a wide range of valuable paradigms that promote the use of AI and Human Movement Analysis in different application domains and at the same time provide rich material for scientific thinking.
